# Using Extended-Release Injectable Aripiprazole for the Successful Treatment of Depressive Symptoms in Bipolar I Disorder

**DOI:** 10.1155/2020/2615748

**Published:** 2020-02-21

**Authors:** Joseph Kuo, Shih-Ku Lin

**Affiliations:** ^1^Department of Psychiatry, Camillians Saint Mary's Hospital Luodong, Yilan, Taiwan; ^2^Taipei City Hospital and Psychiatric Center, Taipei, Taiwan; ^3^Department of Psychiatry, School of Medicine, Taipei Medical University, Taipei, Taiwan

## Abstract

Extended-release injectable (ERI) aripiprazole is indicated for schizophrenia and maintenance monotherapy of bipolar I disorder. Clinical trials of aripiprazole failed to exhibit efficacy in the treatment of bipolar depression. It has been suggested that relatively high doses, rapid titration of dose, a high dropout rate, and a high placebo effect might be the reasons of its ineffectiveness. Here, we report a case of a 39-year-old woman with bipolar depression who was successfully treated with ERI aripiprazole.

## 1. Introduction

Aripiprazole is indicated for the treatment of bipolar disorder, yet it failed to exhibit efficacy for bipolar I depression in 2 multicenter, randomized, double-blind, placebo-controlled studies [1]. It has been suggested that some reasons might be related such as relatively high doses (on average, 15.5 and 17.6 mg/d for each study) administered, rapid titration of dose, a high dropout rate, and a high placebo effect. Another randomized placebo-controlled pilot study also concluded that adjunctive aripiprazole does not seem to be a promising strategy for the acute treatment of bipolar depression [2]. While in a study on bipolar II or bipolar not otherwise specified as depression, low doses of aripiprazole—5 mg or less—were more effective underwent off-on-off-on trials experienced statistically significant improvement [3]. Extended-release injectable (ERI) aripiprazole is also indicated for schizophrenia and maintenance monotherapy of bipolar I disorder. Herein, we report a case where bipolar I depression was successfully treated using ERI aripiprazole once-monthly for 300 mg.

## 2. Case Report

A married 39-year-old native woman lived in the mountain area, distant from the town. She had no previous history of medical or other psychiatric disorder and no definite feature of personality disorder. She had first 3 manic episodes approximately 20, 10, and 8 years ago, respectively. They subsided after approximately a month of drug treatment with combined valproic acid with risperidone or olanzapine while admitted to a hospital. Her first depressive episode was noted soon after the second manic episode, and it subsided approximately 1 to 2 months later after treatment with the combination of valproic acid 1000 mg and bupropion 150 mg/d. In the first 2 instances, she quit all drug treatment soon after her mood symptoms subsided, but after the third manic episode, she exhibited good drug compliance—specifically, she took valproic acid 1000 mg/d for nearly 2 years. The fourth manic episode happened approximately 3 years ago, and it also subsided after treatment following admission. Thereafter, she stopped all drug treatment soon after being discharged. Consequently, severe depressive symptoms, including depressed mood, hypotalkativeness, loss of interest/pleasure, lack of motivation, poor concentration, poor appetite with prominent body weight loss, anergia, hypersomnolence, poor housekeeping, and complete social withdrawal, began approximately 2 years ago. The depressive symptoms persisted for over 1 and half year because she insistently refused all oral drugs and other treatment modalities, including admission and any form of psychotherapy. Normally, administration of oral medication or acute formulas of antipsychotics should be tried before applicating ERIs, but she also refused trial by oral form of aripiprazole and no short-acting aripiprazole injection was available in Taiwan. Besides, measurement of serum levels of aripiprazole was not possible in the hospital. After some persuasion, she agreed that we could administer her first intramuscular injection of aripiprazole once-monthly for 300 mg by home treatment program. Unexpectedly, the aforementioned severe depressive symptoms completely subsided 1 month later and no definite drug side effect was found. Her score on the 17-item Hamilton Depression Rating Scale decreased from 40 to 4. She could competently perform housekeeping tasks and tried to find a full-time job. She was motivated to receive continuous treatment and her symptoms continued to be remitted after 6 shots of aripiprazole once-monthly for 300 mg.

## 3. Discussion

Earlier reports indicated that aripiprazole could be helpful for both major depression and bipolar depression. However, it had a high discontinuation rate, primarily due to side effects [4, 5]. A post hoc analysis of the combined randomized, double-blind, placebo-controlled studies for bipolar I depression also indicated that lower doses of aripiprazole may be more effective [6]. Therefore, the efficacy of aripiprazole for treating bipolar depression remains inconclusive. In this case, the woman had a prolonged, persistent course of depression and refused any kind of oral drug treatment or treatment modality. Though no trial of oral form of aripiprazole was performed, it was thus rational to have tried aripiprazole once-monthly at a relatively low dose of 300 mg to reduce the possibility of side effects. It worked, and she recovered and was able to take good care of her family. However, the efficacy and tolerability of aripiprazole once-monthly for 300 mg for bipolar depression still require future randomized, double-blind, placebo-controlled studies.
